# New Trends in Biosurfactants: From Renewable Origin to Green Enhanced Oil Recovery Applications

**DOI:** 10.3390/molecules29020301

**Published:** 2024-01-06

**Authors:** Dilshad Shaikhah, Valeria Loise, Ruggero Angelico, Michele Porto, Pietro Calandra, Abraham A. Abe, Flaviano Testa, Concetta Bartucca, Cesare Oliviero Rossi, Paolino Caputo

**Affiliations:** 1 Institute of Functional Surfaces, School of Mechanical Engineering, University of Leeds, Woodhouse Lane, Leeds LS2 9JT, UK; d.m.shaikhah@leeds.ac.uk; 2 Scientific Research Centre, Soran University, Erbil 44008, Kurdistan Region, Iraq; 3 Department of Chemistry and Chemical Technologies, University of Calabria, Via P. Bucci Cubo 14D, 87036 Rende, CS, Italy; valeria.loise@unical.it (V.L.); concetta.bartucca@unical.it (C.B.); cesare.oliviero@unical.it (C.O.R.); paolino.caputo@unical.it (P.C.); 4 Department of Agricultural, Environmental and Food Sciences (DIAAA), University of Molise, Via De Sanctis, 86100 Campobasso, CB, Italy; 5 National Research Council, CNR-ISMN (National Research Council-Institute for the Study of Nanostructured Materials), Strada Provinciale 35D n.9–00010, 00010 Montelibretti, RM, Italy; pietro.calandra@cnr.it; 6 Department of Chemistry, University of Bari, Via E. Orabona 4, 70126 Bari, BA, Italy; abraham.abe@uniba.it; 7 Department of Computer Engineering, Modeling, Electronics and Systems Engineering, University of Calabria, Via P. Bucci Cubo 45A, 87036 Rende, CS, Italy; f.testa@unical.it

**Keywords:** enhanced oil recovery, biosurfactants, microbes, interfacial tension, wettability alteration, emulsion, selective plugging, bitumen, hydrophobic-lipophilic difference

## Abstract

Enhanced oil recovery (EOR) processes are technologies used in the oil and gas industry to maximize the extraction of residual oil from reservoirs after primary and secondary recovery methods have been carried out. The injection into the reservoir of surface-active substances capable of reducing the surface tension between oil and the rock surface should favor its extraction with significant economic repercussions. However, the most commonly used surfactants in EOR are derived from petroleum, and their use can have negative environmental impacts, such as toxicity and persistence in the environment. Biosurfactants on the other hand, are derived from renewable resources and are biodegradable, making them potentially more sustainable and environmentally friendly. The present review intends to offer an updated overview of the most significant results available in scientific literature on the potential application of biosurfactants in the context of EOR processes. Aspects such as production strategies, techniques for characterizing the mechanisms of action and the pros and cons of the application of biosurfactants as a principal method for EOR will be illustrated and discussed in detail. Optimized concepts such as the HLD in biosurfactant choice and design for EOR are also discussed. The scientific findings that are illustrated and reviewed in this paper show why general emphasis needs to be placed on the development and adoption of biosurfactants in EOR as a substantial contribution to a more sustainable and environmentally friendly oil and gas industry.

## 1. Introduction

Petroleum, also known as crude oil, is a vital raw material in the chemical industry, playing a significant role in global economic development for the past century [1]. It is a primary energy source for transportation, power generation, and a range of industrial and household applications. The estimated daily oil consumption is about 90 million bbl of oil [2], and it is projected to continue until 2030 with an annual increase of 1.7% in the number of oil barrels produced annually [3,4,5]. With the continuous worldwide increase in energy demand, it is crucial to develop new alternatives, such as wind, solar, nuclear energy, and biomass-converted products, to reduce reliance on fossil fuels [6,7,8,9]. However, although the energy transition favors the use of renewable sources, oil and natural gas will remain the main source of energy for the next decades.

Although several technologies for alternative energy sources are under development, it will take a few decades until global reliance on petroleum and fossil fuels becomes a thing of the past. A lot of research is ongoing in this regard and, according to [10], energy sources capable of completely replacing petroleum are being projected to be in place by the year 2050 at the latest. In order to optimize the availability and use of crude oil and petroleum products, it is essential to improve oil recovery processes by using more environmentally friendly techniques to extract oil from subsurface reservoirs [11,12]. As the reservoirs run out during primary recovery, oil recovery becomes increasingly difficult, even for partially emptied shales or other tight deposits. The oil recovery process involves three stages: primary, secondary, and tertiary recovery. The primary and secondary recovery processes use conventional technologies, including natural energy, water/gas injection, and gas injection, to extract crude oil from the reservoirs [13,14,15,16,17,18]. Several studies show that the primary process only recovers 10–20% of the total oil in the reservoirs.

After the depletion of natural energy, the secondary recovery involves water/gas injection into the reservoir through the injection wells to increase oil displacement toward the producing wellhead. Consequently, the final recovery of crude oil rarely exceeds 50% of the oil originally present. Nonetheless, after the primary and secondary recovery processes, more than two-thirds of the original crude oil remains trapped due to factors such as high viscosity, reduced mobility, and the retention of oil in the pores of rocks [19,20].

To increase the final oil recovery from the reservoirs, tertiary recovery processes, known as enhanced oil recovery (EOR), are applied. Hence, a higher percentage of crude oil recovery can be achieved by lowering the crude oil’s viscosity, improving its flow properties, and modifying the rock wettability, interfacial tension (IFT), and capillary forces.

Among EOR processes, chemical recovery typically utilizes conventional surfactants such as polyoxyethylene alkyl ethers (AE), sodium dodecyl sulfate (SDS), alkyl sulfates (AS), alkyl benzene sulfonates (ABS), alkylpolyoxyethylene sulfates (AES), and polyoxyethylene alkylphenyl ethers (APE) [21]. Despite their effectiveness, these petroleum-based molecules are often non-biodegradable and pose a threat to the environment [22,23]. To address this issue, research efforts have shifted toward identifying biodegradable and bio-based alternatives for surfactants in the oil recovery process. The oil recovery process is crucial to meeting the world’s energy demand and while alternative energy sources are under development, petroleum remains a vital energy source. Therefore, enhancing oil recovery processes, particularly through the use of environmentally friendly technologies based on biosurfactants, is critical for ensuring a sustainable energy future.

Biosurfactants could be a viable option to recover oil from reservoirs in an eco-friendly way as surfactants from natural sources and bio-oils in general have been shown to have several diverse eco-friendly applications in research and industry [24,25,26]. In light of this, attempts to produce biosurfactants derived from natural, renewable sources have been very intensive in the last decade.

In this review, we will first provide an exhaustive overview of the main techniques that use bio-renewable species currently employed to enhance oil recovery (Section 2); then, Section 3 will report a systematic classification of various classes of biosurfactants and the advantages over synthetic surfactants applied in EOR; Section 4 will deal with the strategies of production of biosurfactants and application methods in EOR; in Section 5 the main experimental techniques used to characterize the mechanism of action of biosurfactants will be described in detail; Section 6 will discuss pros and cons of biosurfactant application in EOR and, finally, a comprehensive exposition of future challenges and concluding remarks will be outlined in Section 7.

## 2. EOR Techniques

**Figure 1:**
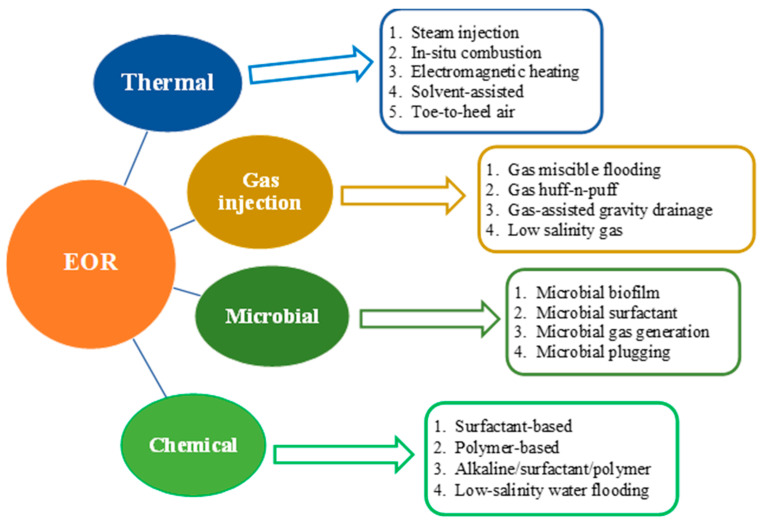
Main methods of enhanced oil recovery.

EOR processes involve various methods, including thermal, chemical, gas and microbial methods, which are listed in Figure 1. In the following subsections, solely the chemical and microbial techniques are revisited critically.

### 2.1. Chemical EOR

Chemical EOR is a technique that involves the injection of chemicals into the reservoir to increase the amount of oil that can be extracted. The chemicals can change the physical and chemical properties of the reservoir rock and fluids, reducing the oil viscosity and improving oil mobility. The different types of techniques employed in chemical EOR are described below.

#### 2.1.1. Surfactant-Based EOR

**Figure 2:**
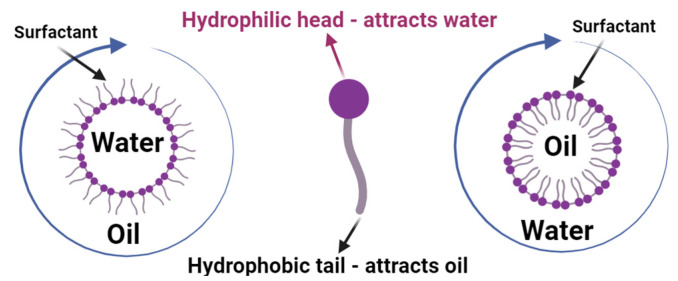
General representation of a surfactant molecule with hydrophilic head and hydrophobic tail and their rearrangement in water and oil.

Surfactants are the most commonly used chemicals in surfactant-based EOR. Owing to the presence of both a hydrophilic polar head and one or two hydrophobic tails (Figure 2), these chemicals can be used to lower the interfacial tension between oil and water, improve the wettability of the reservoir rock, and mobilize the residual oil. However, the effectiveness of surfactants in EOR is limited by factors such as surfactant adsorption, salinity, and temperature. To address these limitations, researchers have developed novel surfactants and surfactant formulations that are more effective in EOR applications [18].

Indeed, due to the environmentally toxic nature of a significant number of synthetic surfactants, research has drifted towards the development of surfactants of low environmental impact which are derived from bio-based sources. Most of these biosurfactants are more eco-friendly than conventional surfactants and also cost-effective in some cases. The cost-effectiveness of biosurfactants depends on the starting material or substrate needed for their production. In most cases, the substrate or starting material is found freely in nature or exists in the form of waste or biomass derived from different processes. However, in some cases, biosurfactant production could require expensive raw material or substrates/feedstock, specialized equipment, and/or high levels of expertise and technical know-how. In previous years, biosurfactant production was not so economically effective, as the cost per kilogram of large quantities of low-value biosurfactants, mostly derived from vegetable oils and highly processed biomass, ranged between 1.5 and 2.5 euros, which is about 20% more than the cost of conventional surfactants [27]. In recent years, however, the cost of biosurfactant production is being alleviated with the advent of several strategies for feasible commercial biosurfactant production such as the use of industrial and agro-based waste and other low-cost renewable substrates [28], genetic recombination and mutation of the starter culture/substrate [29], and the in situ microbial production of biosurfactants in oil reservoirs where they can enhance oil recovery, making the process economically viable [30]. Scientific attempts to produce bio-based surfactants have produced promising results and some biosurfactants capable of replacing conventional chemical surfactants now exist for use in EOR. Several studies conducted by different investigators, such as Li et al. [31], Haghighi & Firozjaii [32], Wu et al. [33], Haq [34], have demonstrated that biosurfactants have been quite effective in EOR applications. Biosurfactants can be obtained from a variety of natural, bio-based, renewable sources. These include microorganisms, animals and parts of plants such as seeds, roots, leaves, natural oils extracted from plants [35,36,37], and waste vegetable oils. Vegetable oils contain triglyceride fatty acid residue, which, when reacted with an alcohol (either methanol or ethanol), produces fatty acid methyl or ethyl esters depending on which alcohol is used [38].

Biosurfactants can also be derived from amino acids which can be obtained from plant and animal sources. The ability of these amino acids to be modified according to the required application makes them very valuable for the production of surfactants [39].

Just like their synthetic counterparts, bio-based surfactants can be classified into anionic, cationic, non-ionic, and zwitterionic surfactants. Two other classes of bio-based surfactants exist: bio-based Gemini surfactants and bio-based polymeric surfactants derived from bio-oils.

- *Bio-based anionic surfactants*. These are surfactants with a negatively charged head and they are the most widely used group of surfactants; hence, they are produced in large quantities in industries. Amino acids and fatty acids obtained from natural oils are viable sources of anionic surfactants. Methyl and ethyl ester sulfonates are derived from these amino and fatty acids via trans-esterification and sulfonation reactions [40]. The sulfonate group (${SO}_{3}^{-}$) present in the chemical structure of these surfactants is responsible for their anionic nature and thermal stability [41]. Another class of anionic amphiphiles can be obtained by linking one or two hydrophobic chains to the DNA nucleotides, giving them the ability to generate supramolecular colloidal structures [42,43,44,45].
- *Bio-based cationic surfactants*. These are surfactants with a positively charged headgroup, and although they are not commonly used in EOR due to their high adsorption in sandstone reservoirs, they are very useful in carbonate reservoirs. Although many plant extracts are considered to be non-ionic, some plant extracts, such as mulberry leaves, olive leaves, and henna leaves, are examples of cationic surfactants [46].
- *Bio-based non-ionic surfactants*. These are surfactants that have no charge on their head. They do not ionize in water, and their solubility is influenced by hydrogen bonds and van der Waal interactions. Most non-ionic surfactants have a high biodegradability and are very cost-effective. Saponins, which are triterpenic or steroidal glycosides, are the most common type of non-ionic surfactant, and they can be obtained from natural plant extracts. They are highly emulsifiable, having excellent solubility and foaming properties, which gives them a wide range of applicability in industrial processes [46,47,48]. Alkyl polyglucoside is a common non-ionic surfactant from a natural and renewable source. *Ziziphus Spina-christy* and soap nut saponin are excellent sources of natural non-ionic surfactants, which have been evaluated for their potential in EOR applications [49,50].
- *Bio-based zwitterionic surfactants*. These surfactants contain both positively and negatively charged residues in their head polar groups having zero net charge. They are very versatile, and natural zwitterionic surfactants have excellent properties that are ideal for EOR applications. For example, a zwitterionic surfactant derived from castor oil, N-phenyl-fattyamido-propyl-N, N-dimethyl-carboxyl betaine (CPDB) has been shown to have excellent thermal properties, dispersion efficiency, optimal wetting and foaming performance and also strong electrolyte tolerance [51]. Despite the versatility and compatibility of zwitterionic surfactants, they are one of the least applied for EOR operations. This is mostly associated with the high costs involved in their production. More work has to be performed to develop cheaper base materials for the synthesis of zwitterionic surfactants and apply this class of surfactants in EOR contexts.
- *Polymeric bio-based surfactants*. They can be synthesized either by polymerizing a surface-active monomer or by the copolymerization of hydrophobic and hydrophilic monomers. Although polymeric surfactants, in most cases, perform poorly regarding surface tension modification—making it difficult to obtain ultra-low IFT values—they could improve oil recovery by combining the high viscosity property of a polymer with the interfacial surface properties of the surfactant present within its structure. This unique combination of properties makes polymeric surfactants ideal for use as thickening agents, IFT-reducing agents, and emulsifying agents for EOR applications [52,53]. A classic example of a polymeric biosurfactant is emulsan, which is produced by *Acinetobacter calcoaceticus* [54].
- *Bio-based Gemini surfactants*. Just like zwitterionic surfactants, bio-based Gemini surfactants are another underused group of bio-based surfactants. They have excellent properties that make them optimal for EOR purposes but ironically, not many research studies investigating their EOR potential are found in the scientific literature. They are very unique in their nature as they are made up of two or more hydrophilic groups, which constitute the head, one hydrophobic group, which makes up the tail, and a spacer linking these two constituents (head and tail). The hydrophilic head could be either anionic, cationic, zwitterionic, or non-ionic [55,56,57]. This means that Gemini surfactants are a sort of hybrid of all the aforementioned classes of bio-based surfactants. This class of surfactants has excellent wetting, solubility, and foaming properties coupled with an ultra-low critical micelle concentration (CMC) and Krafft point [57]. Gemini surfactants can be obtained from amino acids, oils, and sugar [58].

#### 2.1.2. Polymer-Based EOR

Polymers are used in polymer-based EOR to increase the viscosity of the injected water, which helps displace the oil in the reservoir. The effectiveness of polymer flooding is influenced by factors such as polymer concentration, injection rate, and reservoir heterogeneity. To improve the performance of polymer flooding, researchers have explored the use of nanoparticles, smart polymers, and other novel materials [59].

#### 2.1.3. Alkaline/Surfactant/Polymer (ASP) Flooding

ASP flooding is a combination of surfactant, polymer, and alkali flooding techniques. The surfactants are used to reduce the interfacial tension between oil and water, the polymer is used to increase the viscosity of the injected water, and the alkali is used to reduce the acidity of the reservoir rock and improve the wettability. ASP flooding has shown promise in reducing oil viscosity and improving oil recovery, but its effectiveness is influenced by factors such as the choice of chemicals, reservoir characteristics, and operational parameters [60].

#### 2.1.4. Low-Salinity Water Flooding

Low-salinity water flooding involves the use of water with reduced salinity to displace the oil in the reservoir. The low-salinity water can change the wettability of the reservoir rock and reduce the residual oil saturation. However, the effectiveness of low-salinity water flooding is influenced by factors such as the reservoir temperature, salinity, and mineralogy [61].

#### 2.1.5. Critical Features of Chemical EOR

While chemical EOR has shown some promise and has been successfully implemented in certain cases, there are several critical aspects that still need to be considered:

1. *Economic feasibility*. Chemical EOR methods often demand substantial initial investments for the acquisition and injection of chemicals, as well as infrastructure modifications. Evaluation of economic viability becomes paramount, taking into account oil prices, field characteristics, and project lifespan.
2. *Environmental impact*. The substantial use of chemicals in the process can lead to adverse environmental consequences. Toxicity and improper handling risks must be addressed, along with considerations of energy consumption and associated greenhouse gas emissions.
3. *Geologic and reservoir constraints*. Geological and reservoir characteristics, such as permeability, heterogeneity, and natural fractures, profoundly influence the effectiveness of chemical EOR. In-depth reservoir property understanding and rigorous laboratory testing are prerequisites for large-scale implementation.
4. *Chemical optimization*. Challenges often arise from chemical compatibility and precise composition optimization. Incompatibilities can lead to precipitation or emulsion formation, reducing efficacy. Optimizing the chemical composition and concentration is pivotal for maximum recovery with minimal side effects.
5. *Uncertainty and risk*. Inherent uncertainties, including reservoir heterogeneity, fluid behavior, and chemical reactions, pose risks to the success of chemical EOR. Rigorous risk assessment and contingency planning are crucial to mitigate potential setbacks.

In essence, while chemical EOR shows promise, its success hinges on comprehensive evaluation encompassing economic, environmental, geological, and chemical aspects, along with vigilant risk mitigation.

### 2.2. Microbial EOR

Microbial EOR (MEOR) is a technique that involves the injection of microorganisms into the reservoir to enhance oil recovery. The microorganisms can alter the physical and chemical properties of the reservoir, reducing the oil viscosity and improving oil mobility. The first ever field test using MEOR was performed in Arkansas, United States in 1954 when *Clostridium acetobutylicum* was injected alongside molasses into an oil field and the results showed the production of various metabolites such as gases, acids and biosurfactants [62]. Strappa et al. [63] also reported a 20% increase in crude oil recovery yield when they injected facultative anaerobic bacteria along with their nutrients into an oil field. A similar study was carried out by Davidson and Russell [64] by the injection of a specially adapted strain of *Clostridium*, which brought about a reduction in oil viscosity due to the production of carbon dioxide, and this improved oil mobility and sweep efficiency. In China, an incremental production of 219,000 tons of crude oil was reported when microbial huff and puff was carried out on 1640 oil wells [65]. Table 1 groups the products of microbial activity according to the microorganisms that produce them and the type of oil reservoirs they are suitable for, while Table 2 classifies the microorganisms according to their products and the effects they bring about in the oil reservoirs. The techniques used in microbial EOR are described as follows [66].

#### 2.2.1. Microbial Biofilm Injection

In microbial biofilm injection, a microbial biofilm is injected into the reservoir to improve oil recovery. The biofilm can alter the permeability of the reservoir rock and improve oil mobility. However, the effectiveness of microbial biofilm injection is influenced by factors such as the reservoir temperature, salinity, and nutrient availability [67].

#### 2.2.2. Microbial Surfactant Injection

In microbial surfactant injection, microorganisms are used to produce surfactants that can reduce the interfacial tension between the oil and water phases in the reservoir. The reduced interfacial tension can improve oil mobility and enhance oil recovery. Several studies [68,69,70] have been carried out and their results demonstrate the applicability of microbial surfactant injection techniques in field trials. Although microbial surfactant injection and MEOR, in general, have achieved a reasonable amount of success, a study carried out in an offshore field trial in Norway showed negative results when nitrate-reducing bacteria with compatible nutrient support were used for EOR purposes [71]. However, the effectiveness of microbial surfactant injection is influenced by factors such as microbial strain, surfactant production rate, and reservoir conditions [72].

#### 2.2.3. Microbial Gas Generation

In microbial gas generation, microorganisms are used to produce gases such as methane and carbon dioxide that can enhance oil recovery. The gases can reduce the oil viscosity and improve oil mobility. Gao [65] reported the successful application of the microbial huff and puff technique in several field trials in the Shengli and Daqing oil fields in China over the years. However, the effectiveness of microbial gas generation is influenced by factors such as the microbial strain, gas production rate, and reservoir environment [73].

#### 2.2.4. Microbial Plugging

In microbial plugging, microorganisms are used to block high-permeability channels in the reservoir and divert the flow of water and gas to low-permeability regions. The technique can improve oil recovery by increasing the oil saturation in the low-permeability regions. This method was employed to great effect in a field trial in Brazil in 2010 where microbial EOR was carried out in five wells in an onshore field to plug high permeable zones in the reservoir by producing biomass and biopolymer [74]. However, the effectiveness of microbial plugging is influenced by factors such as the microbial strain, injection rate, and reservoir heterogeneity [75].

**Table 1:** Classification of microbial bio-products for EOR, their producing organisms, and the various types of oil reservoirs/formations in which they are applied. Adapted from [73].

Microbial Product Class	Microorganisms and Their Sample Products		Type of Oil Reservoir/Formation
Biosurfactants	Surfactin	*Rhodococcus sp.*	Sandstone or carbonate reservoirs with moderate temperature (<50 °C) and relatively light oil (API > 25)
	Rhamnolipid	*Acinetobacter*	
	Emulsan	*Bacillus sp.*	
	Lichenysin	*Bacillus sp.*	
	Alasan	*Pseudomonas*	
	Viscosin	*Arthrobacter*	
Biopolymers	Xanthan gum	*Xanthomonas sp.*	Stratified reservoirs with permeable zones
	Pullulan	*Aureobasidium sp.*	
	Levan	*Bacillus sp.*	
	Curdlan	*Alcaligeness sp.*	
	Dextran	*Leuconostoc sp.*	
	Scleroglucan	*Sclerotium sp.*	
Gases	CO_2_	Fermentative bacteria	Heavy-oil-bearing formations (API < 15)
	CH_4_	Methanogens	
	H_2_	*Clostridium*	
	N_2_	*Enterobacter*	
Acids	Propionic acid	Fermentative bacteria	Carbonate or carbonaceous reservoirs
	Butyric acid	*Clostridium*	
Alcohol/Solvents	Alcohols and Ketones (co-surfactants)	Fermentative bacteria	Heavy-oil-bearing formations (API < 15) and strongly oil-wet, waterflooded reservoirs
	Acetone	*Clostridium*	
	Butanol	*Zymomonas*	
	Propan-2-diol	*Kliebsella*	

The effects conferred by the different groups of microbial bio-products are listed in Table 2.

**Table 2:** Microbial EOR classification based on the types of microorganisms, their products, and their effects in the oil reservoirs. Adapted from [73,76,77,78].

Microorganism Genus	Products	Effect
*Pseudomonas*	Surfactants and polymers	Production of biopolymers and biosurfactants, which reduce permeability and enhance capillary number.
*Clostridium*	Gases, acids, alcohols, and surfactants	Production of acid and gases, which reduce oil viscosity.
*Bacillus*	Acids and surfactants	Production of gases, alcohol, and biosurfactants, which modify permeability, which improves sweep efficiency in waterflooding processes.
*Desulfovibrio*	Gases and acids	Oil biodegradability and viscosity reduction along with methane production.
*Corynebacterium*	Surfactants	Production of low-viscosity molecules and permeability modification by promoting oil biodegradability.
Others	Polymers, gases, surfactants, acids, and alcohol	Oxidation and biodegradability of hydrocarbons, permeability modification, and methane production, which lead to oil viscosity reduction.

#### 2.2.5. Critical Features of Microbial EOR

While MEOR shows potential as an environmentally friendly and cost-effective method, there are several essential parts that require attention:

1. *Efficacy and reliability*. MEOR’s effectiveness varies based on microbial strains, reservoir conditions, and oil type. Microorganism growth is sensitive to factors like temperature, pH, and nutrient availability. Strain selection and reliability necessitate thorough evaluation via field testing and case studies;
2. *Reservoir compatibility*. MEOR may not suit all reservoir types due to factors like permeability, heterogeneity, and oil properties. A rigorous assessment of microorganism-reservoir compatibility is essential to determine MEOR applicability;
3. *Long-term effects and sustainability*. The enduring impacts of introducing microorganisms into the reservoir require further understanding. Microbial activities can influence permeability, fluid behavior, and geochemical reactions, necessitating an evaluation of risks and effects on reservoir integrity and oil recovery sustainability;
4. *Regulatory compliance*. MEOR involves the introduction of living organisms into the reservoir, which may raise regulatory concerns. It is important to comply with relevant environmental regulations and obtain necessary permits for the use of microorganisms in oil reservoirs. Additionally, potential risks associated with the release of genetically modified organisms (GMOs) should be carefully assessed and addressed in accordance with applicable regulations and guidelines;
5. *Implementation challenges*. Specialized equipment, ideal growth conditions, and the management of risks such as biofouling and corrosion present implementation challenges. Proper engineering design, operational protocols, and monitoring strategies are essential for successful MEOR implementation;
6. *Knowledge gaps and research requirements*. Despite advancements, significant knowledge gaps persist. Further research is vital to improve microbial strain selection, enhance reservoir suitability assessment, and understand MEOR mechanisms, long-term sustainability, and optimization.

Overall, MEOR presents a potentially environmentally friendly and cost-effective EOR method. However, validation through research, testing, and regulatory compliance, along with thorough evaluation and continuous monitoring, is imperative for its successful and sustainable implementation.

### 2.3. A Brief Comparison: Chemical–Microbial and Traditional EOR Techniques Employed

Chemical–microbial EOR techniques are a subset of EOR methods that utilize both chemicals and microbes to enhance oil recovery. These techniques are highly effective due to their ability to reduce interfacial tension, increase sweep efficiency, and alter wettability, which are key factors in improving oil recovery [79,80,81]. However, the implementation of these techniques often involves higher costs. This is primarily due to the price of the chemicals and microbes used, as well as the need for sophisticated injection equipment [82,83].

On the other hand, thermal and gas injection methods are other types of EOR techniques that can be more cost-effective, although their suitability can vary depending on reservoir conditions [84,85,86]. Thermal recovery methods typically involve the use of heat, often in the form of steam generated by burning natural gas, to reduce oil viscosity and improve its flow. Solar-generated steam in EOR is another method that uses concentrating solar power technology to produce steam. Gas injection methods, which serve as a main EOR method in fractured-vuggy carbonate reservoirs, involve injecting gases like carbon dioxide or nitrogen into the reservoir to increase pressure and displace oil [87]. In terms of environmental impact, chemical-microbial EOR methods can pose challenges due to potential groundwater contamination. However, these risks can be mitigated with proper management and the use of environmentally friendly chemicals and microbes [82,83]. Thermal EOR methods also face environmental challenges, such as cyclic fluctuations in steam injection rate associated with sunlight hours and seasonal variations, which challenge this technology from becoming a standalone solution [84]. Gas injection methods have been shown to provide major contributions during EOR, including crude-oil viscosity reduction, thermal expansion, and crude oil vaporization [87].

While chemical-microbial EOR techniques offer high efficiency in oil recovery, they also involve considerations such as cost and environmental impact. Therefore, it is crucial to choose the most suitable EOR technique based on reservoir conditions and economic feasibility.

## 3. Biosurfactants: Nature, Properties, and Their Applications to EOR

Biosurfactants are surface-active compounds produced from bio-based sources which include plants, animals, and microorganisms, such as bacteria, fungi, and yeasts. Studies such as [33,49,63] have demonstrated the potential of surfactants from bio-based sources for use in EOR applications. These amphiphilic molecules possess both hydrophilic (water-loving) and hydrophobic (oil-loving) regions within their structure, allowing them to reduce the interfacial tension between oil and water, leading to improved oil mobility and displacement in reservoirs [88,89].

### 3.1. Classes of Microbial-Based Biosurfactants Commonly Used in EOR

**Figure 3:**
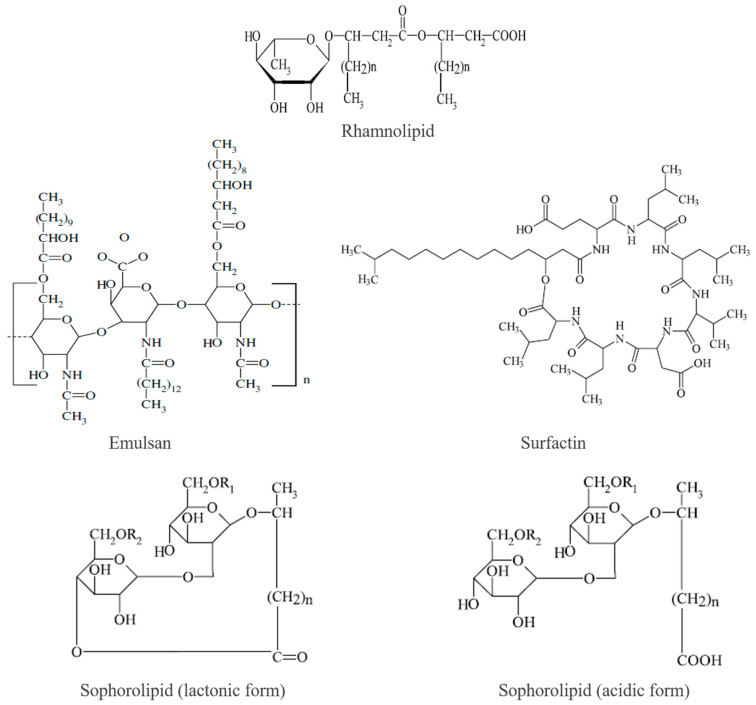
Chemical structures of common microbial-based biosurfactants.

Microorganisms are one of the sources of biosurfactants and different microorganisms have the genetic capability to produce specific types of biosurfactants, resulting in a wide range of compounds with diverse properties [90]. There are several classes of compounds which bring about different biosurfactants. These classes are described below and their examples are shown in Figure 3.

- *Glycolipids*. Glycolipids are biosurfactants composed of a hydrophilic carbohydrate moiety (e.g., glucose, rhamnose) linked to a hydrophobic fatty acid chain. They are produced by various microorganisms, including bacteria and yeasts. Glycolipids have shown excellent surface activity, high emulsification capacity, and stability over a wide range of environmental conditions. Examples of glycolipids used in EOR include sophorolipids and rhamnolipids. Rhamnolipids are biosurfactants composed of one or two rhamnose sugar units linked to a fatty acid chain. They are predominantly produced by *Pseudomonas aeruginosa*, a common bacterium found in various environments. Rhamnolipids have excellent surface tension reduction properties and emulsification capacity. They are known for their high biodegradability and low toxicity, making them environmentally friendly options for EOR applications [91].
- *Lipopeptides*. Lipopeptides are biosurfactants characterized by a cyclic or linear peptide structure linked to a fatty acid chain. They are mainly produced by bacteria, such as *Bacillus* species. Lipopeptides exhibit strong surface activity, foam-forming capability, and suitable stability. Surfactin, produced by *Bacillus subtilis*, is a well-known lipopeptide used in EOR due to its emulsification properties and ability to reduce interfacial tension [92]. Lichenysin produced by *Bacillus licheniformis* is also another example of this class of microbial biosurfactant [54].
- *Lipopolysaccharides (LPS)*. Lipopolysaccharides are complex biosurfactants composed of lipids and polysaccharides. They are typically produced by Gram-negative bacteria, such as *Pseudomonas* and *Serratia* species. LPS exhibit strong surfactant activity and have the ability to form stable emulsions. They also possess immunostimulatory properties, which can impact their application in EOR [93].
- *Phospholipids*. Phospholipids are a class of biosurfactants that consist of a hydrophilic phosphate head group and two hydrophobic fatty acid tails. They are abundant in the cell membranes of microorganisms, including bacteria and yeasts. Phospholipids have been investigated for their ability to reduce interfacial tension and improve oil recovery efficiency. However, their high cost and limited production scale have limited their widespread use in EOR [94].

Each type has unique properties and performance characteristics, and their selection depends on factors such as reservoir conditions, desired oil recovery mechanisms, and cost-effectiveness. Continued research and development in biosurfactant production and application are crucial for expanding their use in the field of EOR.

### 3.2. Advantages of Biosurfactants over Synthetic Surfactants Applied in EOR

Biosurfactants, surface-active compounds produced by microorganisms, offer several advantages over synthetic surfactants in the context of EOR. Here are the key advantages:

- *Biodegradability:* Unlike synthetic surfactants, which are typically derived from petrochemicals and may persist in the environment, biosurfactants can be easily broken down by natural microbial processes. This biodegradability reduces the potential for long-term environmental impact and makes biosurfactants a more sustainable choice [95,96].
- *Environmental friendliness:* Biosurfactants have a low ecological footprint compared to synthetic surfactants. They are produced using renewable resources and exhibit lower toxicity levels. This characteristic minimizes the risk of polluting the environment during their production, application, and eventual degradation. Biosurfactants are considered eco-friendly alternatives for EOR operations, aligning with the principles of green chemistry and sustainable practices [97,98].
- *Compatibility with reservoir conditions:* Biosurfactants can be tailored and optimized to suit specific reservoir conditions, such as temperature, salinity, and pH. They often exhibit suitable stability and surface activity over a wide range of environmental parameters. This versatility allows biosurfactants to maintain their effectiveness in challenging reservoir conditions, where synthetic surfactants may be less stable or lose their activity. The ability of biosurfactants to function under harsh conditions enhances their applicability in various oil recovery processes [33].
- *Selectivity and specificity*: Biosurfactants can be engineered to exhibit selectivity for oil–water interfaces, allowing them to target and interact specifically with the oil phase. This selectivity improves the efficiency of oil displacement and recovery, as biosurfactants can preferentially adsorb at the oil–water interface, reducing interfacial tension and facilitating oil mobilization. Synthetic surfactants, on the other hand, may exhibit broader interactions, leading to potential drawbacks such as excessive foam production or unwanted interactions with reservoir minerals [99].
- *EOR potential*: Biosurfactants have shown promising results in enhancing oil recovery efficiency. They can effectively reduce interfacial tension between oil and water, leading to improved oil mobilization and displacement. The unique chemical structures and properties of biosurfactants, including their ability to form stable emulsions, make them valuable agents for enhancing oil recovery from reservoirs [100].

While biosurfactants offer several advantages, their commercial use in EOR is still being explored and faces challenges such as cost-effectiveness, scale-up production, and compatibility with existing field operations. Some of the challenges faced by biosurfactants in EOR, such as life cycle assessment (LCA) challenges and the need for co-surfactants, are explained later on in this review. However, ongoing research and development efforts aim to address these limitations and further enhance the application of biosurfactants in EOR.

### 3.3. Dynamical Aspects in Biosurfactant Action

In the context of biosurfactants in EOR, amphiphilicity, commonly associated with surfactants, manifests in various forms, such as micellar systems, mixtures of pure amphiphiles, block copolymers, and liquid crystals. These diverse self-assembled structures exhibit nano-segregated polar and apolar domains, characterized by locally ordered structures with system-dependent lifetimes. Mean size and lifetime parameters are crucial in controlling macroscopic physicochemical properties, impacting technological applications. Micellar systems, representing biosurfactant-made supramolecular aggregates [101], undergo dynamic processes with distinct timescales:

- Rotation and conformational change of monomers (nanoseconds);
- Lateral diffusion of monomers on a meso-interface (milliseconds);
- Aggregate shape changes and fluctuations;
- Breaking and reforming of micelles or supramolecular aggregates;
- Aggregate collisions, sometimes resulting in fusion events.

The simultaneous presence of different biosurfactants can induce emerging properties, a noteworthy aspect in materials from the crude oil industry or bituminous materials. Interactions between two amphiphilic substances can lead to unique self-assembly and enhanced dynamical properties. For instance, blends involving biosurfactants demonstrated a notable increase in proton conductivity, indicating altered molecular self-assembly and dynamical processes [102,103,104,105]. The interaction dynamics between different biosurfactants, and surfactants in general, introduce factors like steric effects, van der Waals interactions, polar interactions, and H-bond formation, influencing medium-range structure formation. These organized structures may create pathways for specific dynamic processes, offering tailored properties for novel applications.

## 4. Production of Biosurfactants and Application Methods in EOR

Different natural sources, including organic waste materials, can be exploited in a convenient and efficient way to produce various types of surfactants that may be of interest in EOR, while the application methods consist mainly of both in situ and ex situ treatments using biosurfactant-producing microbes.

### 4.1. Biosurfactant Production

Common biosurfactant production strategies are based on the use of microbial, vegetable, and animal sources, as will be outlined below.

#### 4.1.1. Biosurfactants from Microbial Sources

There are several ways in which microbial activity can be exploited in order to produce biosurfactants:

- *Microbial fermentation*. This is one of the most common methods of biosurfactant production. It involves culturing cells in a suitable growth medium such as agars under specific conditions. This cultivation of cells includes the selection of suitable microbial strains, the development of the inoculum, and larger-scale fermentation followed by recovery and purification. *Pseudomonas aeruginosa* and *Bacillus subtilis* are suitable strains for biosurfactant production via fermentation in general [106];
- *Submerged fermentation.* This is also widely used in biosurfactant production. In this method, the microorganisms are grown in a well-aerated liquid cell culture medium, also known as broth. This method improves the microbial yield and is very easy to carry out. The challenge associated with this method, however, is that foam control can prove to be difficult. This method also results in high energy consumption due to agitation and aeration requirements [107];
- *Solid-state fermentation (SSF)*. This is the most green and eco-efficient biosurfactant production method. It involves the cultivation of microorganisms on solid substrates, such as agricultural waste. The firm surface of the solid substrates gives ample surface and conditions for microbial replication and biosurfactant production. There are several advantages associated with SSF, such as cost-effectiveness, end-of-waste (EoW) application via the utilization of waste materials to create a circular economy system, lower energy and water requirements, and so on. A few downsides to the SSF method also exist, such as process control and uniformity coupled with the difficulty in the recovery of biosurfactants from solid matrices [108];
- *Fed-batch fermentation*. This is a method in which nutrients are gradually added during the fermentation. It helps to maintain excellent growth conditions and optimal waste management, bringing about higher yield compared to batch fermentation [106];
- *Genetic engineering*. This biotechnological method involves several techniques that are used to improve the production of biosurfactants by genetically modifying the biosurfactant-producing microbes. It involves knocking-out and inserting specific genes in order to improve biosurfactant synthesis pathways. This opens up the possibility of the use of cheaper alternative substrates, leads to an increase in yield, and also improves the properties of the biosurfactants produced. This technique, however, requires technical know-how in order to modify the genetic makeup of the microorganisms [24].

#### 4.1.2. Biosurfactants from Plant and Animal Sources

Plants and animals are also a viable source of biosurfactants. There are several ways biosurfactants can be obtained from plant and animal sources:

- *Extraction of biosurfactants from plant sources*. Due to the vast array of bioactive compounds that can be found in plants, plant materials such as leaves, seeds, fruits, and roots are often used as sources of materials for biosurfactant production [109]. These materials are subjected to different extraction methods, such as solvent extraction, maceration, and supercritical fluid extraction. Water and organic solvents such as ethanol and methanol are some of the solvents used to extract these bioactive compounds from plants. The solvent and extraction method depends on the plant and the type of surfactant to be produced. The extracted mixture can then be purified using techniques such as membrane filtration, solvent partitioning, and column chromatography [110,111]. Extraction serves to help remove the impurities in order to obtain the desired biosurfactant. Several plant oils and surfactants have been used to great effect in attempts to evaluate their potential to potentially replace harmful chemicals, which are currently used for several purposes in research and industry [112,113] and plant-based surfactants for use in EOR are no exception.
- *Extraction of biosurfactants from animal sources*. Animal tissues, including organs and glands, are also an ideal source of biosurfactants such as lipopeptides. The tissue is first homogenized, and then the biosurfactant can be extracted using solvents or other extraction methods [114]. Animal by-products such as lipids, waste fat and protein can be used as substrates for microbial fermentation to obtain biosurfactants as described in the previous section of this review.
- *Microbial conversion of plant and animal biomass*. As previously described, microorganisms have the ability to convert plant and animal biomass to biosurfactants. These microorganisms are cultivated on plant or animal-based substrates, and they break down the complex compounds in the biomass, producing biosurfactants as by-products [115].

### 4.2. Method of Application of Biosurfactants in EOR

Eco-friendly enhanced oil recovery methods are gaining attention due to their eco-friendly nature, cost-effectiveness, and improved applicability. These methods involve the use of surfactants derived from natural sources such as microorganisms, plants, and animals. In the case of microorganisms, as mentioned earlier in this review, their different metabolite products and growing properties affect the recovery method, leading to EOR. Biosurfactants can be used in EOR using several approaches:

- Injecting cell-cultured biosurfactant-producing microorganisms from wells toward the reservoir and consequent in situ replication and diffusion through the reservoir rocks.
- Ex situ injection of appropriate nutrients into the reservoir to stimulate the growth of biosurfactant-producing microbes already present in the reservoir.
- Production of the biosurfactants externally, which are subsequently injected into the reservoir. This occurs in the case of biosurfactants obtained from waste materials, plant or animal sources, and, in some cases, microbes [116,117].

Glycolipid-based surfactants obtained from *Pseudomonas* strains have been used in MEOR experiments, and lipopolysaccharides and lipopeptides such as lichenysin and surfactin have been found to be quite effective in MEOR, with surfactin being the most promising microbial surfactant so far [96,116]. *Pseudomonas*, *Bacillus*, *Sphingomonas*, and *Actinobacteria* spp. are the most effective biosurfactant-producing bacteria, while saturated and unsaturated fatty acids obtained from plant sources such as *Jatropha curcas*, *Zizyphus Spina-Christi*, *Glycyrrhiza Glabra,* and so on are viable primary materials of ideal biosurfactants suitable for EOR [2,110].

## 5. Role of Biosurfactants in EOR and Techniques for Characterizing Their Performance

Capillary forces play a central role in oil recovery as they directly influence oil displacement from reservoir rocks. These forces exist due to the interfacial tension between oil and water. The use of biosurfactants can enhance oil recovery by overcoming these capillary forces, thereby altering the physicochemical parameters of the system, which then facilitates oil displacement in porous rock formations.

### 5.1. Role and Mechanisms of Action of Biosurfactants in EOR

The role of effective biosurfactants in EOR, in general, is to improve oil recovery yield via the following three mechanisms:

- Reduction in the interfacial surface tension (IFT);
- Alteration of the wettability of an oil-wet reservoir rock;
- Mobilization of the trapped oil via emulsification.

A significantly reduced IFT and modified wettability increase the capillary number, which results in a higher oil recovery. IFT can be described as the adhesive tensional force present between oil and water molecules, which keeps these two phases trapped in the pores of the reservoir rock. In order to improve oil recovery, the capillary force that keeps the oil together must be reduced by lowering the IFT, which results in a higher capillary number, causing the residual oil to flow toward the oil bank and then to the production well point of extraction. Due to the amphiphilic nature of the surfactant, upon introduction of the surfactant into the reservoir rock system, the hydrophilic head attaches to the water/brine while the hydrophobic tail attaches to the oil phase [118].

**Figure 4:**
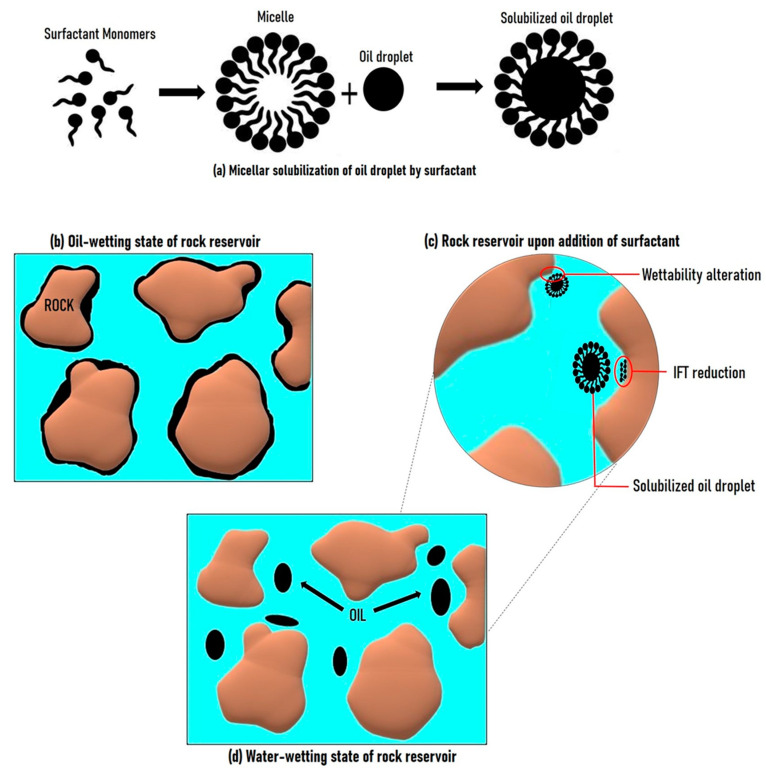
Illustration of the mechanism via which surfactants bring about wettability alteration and IFT reduction in a reservoir rock system during EOR.

This orientation of the surfactants’ head and tail causes a decrease in the system’s free energy, thereby reducing the IFT (see Figure 4). Studies on model systems showed that strong and effective interactions can be established between the surfactant polar heads and the water, even in small amounts of the latter [119]. In order to reduce the IFT, the biosurfactant molecules should be adsorbed unto the surfaces of the two immiscible liquids. The process of adsorption of the surfactant molecules is a dynamic process, and it continues until it reaches an equilibrium state, which is considered to be the final static interfacial tension [120,121]. Critical micelle concentration (CMC) is also an important factor in the reduction of IFT in oil–water systems [122]. The solubility of the surfactant and the salinity of the oil reservoir are also important factors in IFT reduction and wettability alteration [123]. The adsorption of surfactants is highly influenced by salinity. As the salinity increases, so does the surfactant adsorption to the surface of the oil and water phases.

Ions such as Na^+^, K^+^, and Mg^2+^ have been found to influence biosurfactant adsorption on carbonates [124]. It is indeed ascertained that effective interactions between ionic species and the polar head of certain surfactants can take place [125].

Wettability is another important parameter that has to be modified by the biosurfactant in order to improve oil recovery from reservoirs. Wettability is the ability of a liquid to make contact with a solid surface. It is the tendency of a fluid to spread on or adhere to a surface in the presence of other immiscible fluid. A suitable biosurfactant for EOR should be able to modify the wettability of porous media from oil-wetting condition to water-wetting condition, which is desired for improved oil recovery (Figure 4).

By modifying the wettability of the rock substrate to favor water-wetting, the capillary adhesive force that strongly attaches the oil to the rock reduces, improving the flow of the oil [118]. Wettability is a very important factor in EOR because it affects several parameters, such as relative permeability and oil–water saturation. The contact angle measures the degree of the modification of the wettability and is generally used for wettability determination [126]. Nafisifar et al. used the contact angle method to measure wettability alteration in their study [127].

Emulsification is another mechanism via which biosurfactants can enhance oil recovery. Emulsions are thermodynamically unstable systems obtained when two immiscible liquids are mixed by dispersing one into the other. Oil can be dispersed in water, which brings about oil-in-water emulsions, or water can be dispersed in oil, which brings about water-in-oil emulsions. Water-in-oil (W/O) emulsions are more effective in EOR. Emulsions are produced by mechanically breaking the dispersed phase into tiny droplets, which are then dispersed into the continuous phase [128].

Spontaneous emulsification can also occur, and this is dependent on factors such as interfacial turbulence, transient negative values of IFT, and diffusion and stranding via chemical instability [129]. Aside from IFT reduction and wettability modification, surfactants can facilitate the formation of emulsions by increasing the surface viscosity, the surface elasticity, and the electric double-layer repulsion. Suitable surfactants for EOR can also determine which phase (either oil or water) will be the dispersed phase or the continuous phase by their HLB values.

Surfactants with HLB values ranging from 3 to 8 form W/O emulsions, while those with HLB values ranging from 8 to 18 form oil-in-water (O/W) emulsions [130]. Apart from the HLB concept for the classification of surfactants, another concept for the characterization of surfactants used in EOR known as the hydrophilic–lipophilic difference (HLD) has more recently been gaining widespread acceptance and is being increasingly utilized in EOR operations.

### 5.2. Influence of the General Concepts of Hydrophilic–Lipophilic Difference (HLD) and Hydrophilic–Lipophilic Balance (HLB) on the Efficiency of Ionic and Non-Ionic Biosurfactants

The hydrophilic–lipophilic balance and hydrophilic–lipophilic difference of surfactants are two approaches used for characterizing surfactants and their behavior not only in EOR but across a wide range of formulation-based contexts. HLB was developed to classify non-ionic ethoxylated surfactants according to their hydrophilicity on a scale of 0–20. Although HLB has proven to be useful in some cases, the fact that it applies only to non-ionic ethoxylated surfactants limits its predictive power on balanced systems and microemulsions. HLD, on the other hand, is a more in-depth screening method for surfactant–oil–water (SOW) formulations based on the characteristics of the surfactant. The HLD concept involves the use of an equation that takes into account four main parameters in balancing the whole system of water, surfactant, oil, temperature, and salts. The HLD concept is a powerful tool in EOR as the average conditions of rock reservoirs in EOR fit the description of this aforementioned whole system of water, surfactant, oil, temperature, and salts. The HLD concept is capable of determining the characteristic curvature (*Cc*) of a surfactant, and this can be applied to predict the most suitable surfactant structure for an EOR formulation [131]. The four main parameters taken into account by the HLD equation are the effective alkane carbon number (EACN) of the oil, the temperature in °C, the salinity, and the characteristic curvature (*Cc*) of the hydrophobic/hydrophilic nature of the surfactant.

While both HLB and HLD have their merits, there are certain advantages to using HLD over HLB in specific situations, especially in the context of enhanced oil recovery. Some of these advantages include greater flexibility to accommodate a wider range of surfactant systems and conditions, suitability for complex systems with multiple surfactants, co-surfactants, and additives, and a higher degree of predictability for surfactant behavior under different conditions, among others. HLD can also be used in conjunction with mathematical modeling to simulate surfactant behavior in different scenarios. The synthesis and design of biosurfactants for use in EOR has to take HLB and especially HLD into consideration so as to bring this class of green surfactants to the forefront of EOR application and make them viable and practical options for use in EOR operations.

#### 5.2.1. The HLB Concept

The hydrophilic–lipophilic balance (HLB) model, introduced by Griffin in 1949 [132], was initially developed to classify non-ionic ethoxylated surfactants (CNEi) according to their hydrophilicity, using a numerical scale from 0 to 20. The general rule, known as Bancroft’s rule, suggests that hydrophilic surfactants tend to yield O/W emulsions, while hydrophobic surfactants tend to yield W/O emulsions. Thus, the HLB theory proposes that to formulate a W/O emulsion, one should use a surfactant with an HLB number between 3 and 6, while O/W emulsions should be performed with surfactants having an HLB number between 8 and 18. Experimental evidence shows that if a given brine/surfactant(s)/oil(s) system at high surfactant concentration forms an O/W microemulsion (mE), it will form, above the emulsification failure, an O/W emulsion. Similarly, systems forming W/O mE, when mixed with water, give rise to stable W/O emulsions. Balanced systems demulsify very fast. In this respect, the HLB can also be a predictive tool for mE. However, the HLB’s predictive power is low, and the extension of the HLB number to non-ethoxylated surfactants is questionable.

Later, in 1976, Israelachvili et al. introduced the idea of a Critical Packing Parameter (CPP) [133]. This theory rationalizes the type of assemblies of pure surfactants in water, depending on the shape of the surfactant. While the HLB model provides a useful classification system for surfactants, the CPP concept offers a more fundamental basis for understanding the self-assembly of surfactant molecules in water. By considering the shape of the surfactant molecule, the CPP can predict the type of aggregates that will form, allowing for the design of more efficient emulsions and microemulsions. Although the critical packing parameter (CPP) is a truly interesting concept that is valuable for understanding liquid crystal phases in concentrated surfactants, many limitations exist in its application in most practical formulations. CPP is useful only in conditions that require an interest in the behavior of higher concentrations of pure surfactants in water but in the case of formulations with oils and emulsions. This behavior does not really apply [134]. 

Sarmah et al. in 2019 [135] characterized and identified non-ionic surfactants based on their ethoxylation numbers and the correlation with their respective HLB values. They tested non-ionic surfactants with HLB values between 12.3 and 17.8 and their work showed that the ethoxylation number can be correlated to HLB. An increase in ethoxylation number resulted in an increase in HLB value. The indications of their study demonstrated that the higher the HLB values of the surfactant, the more desirable it is for EOR applications. This partially corroborates a chapter in Sheng’s book on EOR [136] which states that to form proper microemulsions during the EOR of oil reservoirs with low salinity, low-HLB surfactants should be used, while in situations of high salinity, high-HLB surfactants should be used. Marqués et al. evaluated the HLB of a trehalose tetra-ester biosurfactant produced from *Rhodococcus erythropolis*. And the findings of their study indicated that this biosurfactant was very effective in forming and stabilizing O/W emulsions [137]. Although Bruheim et al. [138], similarly to Sarmah et al. [135], state that there is a correlation between the HLB values and biodegradation of crude oil (higher HLB brings about more efficient biodegradation), Noordman et al. [139] claim that the HLB range varies with the class of biosurfactant used, such as in the case of rhamnolipids which provide O/W emulsion stability and have HLB values greater than 17. In general, the applicability of the HLB concept has no general consensus, as it only seems to work in the case of ethoxylated non-ionic surfactants and does not take into account other factors such as temperature and equivalent alkane carbon number (EACN), and this fact may lead to varying opinions, ideas, and hypotheses on the HLB concept as a whole.

Ionic surfactants, which are molecules that contain both a hydrophilic and a hydrophobic component, are also widely used in EOR processes. Since the HLB concept is based on the assumption that surfactants are non-ionic and that they do not carry an electrical charge, this presents a limitation when it comes to using the HLB concept to predict the performance of ionic surfactants in EOR. Ionic surfactants have an electrostatic interaction with the reservoir rock and other charged species in the reservoir. This interaction can greatly affect their performance, and it cannot be predicted by the HLB value alone [140]. In general, HLB only works in the case of ethoxylated surfactants. For other types of surfactants, a different concept that takes into account parameters such as the oiliness of the surfactant, temperature, salinity, and water is needed [141].

Another limitation of the HLB concept is that it assumes that the surfactant will only interact with oil and water. However, in reality, surfactants in EOR processes can interact with a wide range of other species, including minerals and gases, which can greatly affect their performance. Therefore, the HLB concept cannot fully account for all of the factors that affect the performance of surfactants in EOR [142,143].

Despite these limitations, the HLB concept can still be useful in the selection and formulation of non-ionic surfactants for EOR applications. Non-ionic surfactants are less affected by the electrostatic interaction with the reservoir rock and other charged species in the reservoir, and their performance is primarily determined by their HLB value. However, even for non-ionic surfactants, the HLB concept should be used as a guide, and other factors such as electrostatic interactions, EACN, temperature, and surfactant characteristic curvature should also be considered when selecting and formulating surfactants for EOR applications [144,145].

#### 5.2.2. The HLD Concept

The hydrophilic–lipophilic *HLD* is a concept that was developed as an alternative to the hydrophilic–lipophilic balance (HLB) for the characterization of surfactants used in EOR operations. The *HLD* concept takes into account the chemical nature of the surfactant and the oil phase, and it has been shown to provide a more accurate prediction of the surfactant behavior at the oil–water interface than the HLB concept. In the 1970s, while investigating the surfactants used in enhanced EOR, the balanced mEs found in a Winsor III phase separation were associated with an equal chemical potential of the surfactant in the excess aqueous ($\mu_{s}^{w}$) and oil ($\mu_{s}^{w}$) phases. Generally, their difference, normalized by thermal energy, is called the hydrophilic–lipophilic difference (*HLD*) [146]:

(1) $$HLD=\frac{\mu_{s}^{w}-\mu_{s}^{o}}{RT}.$$

The chemical potentials can be split into additive contributions accounting for the effects of the oil and surfactant nature, temperature, ionic strength, and so on. Accordingly, a first set of empirical correlation equations was proposed to find the balanced (*HLD* = 0) condition for anionic surfactants:

(2) $$HLD=Cc-k\left(EACN\right)-c(T-25\;{}^{\circ}C)+lnS$$

and for nonionic surfactants [134,147,148]:

(3) $$HLD=Cc-k\left(EACN\right)+c’(T-25\;{}^{\circ}C)+bS$$

The parameter *EACN* is the equivalent alkane carbon number of the oil, which in the case of linear alkanes, corresponds to the number of carbon atoms. *Cc* is the characteristic curvature of the surfactant film. These equations are found to be robust for mE systems. In Equations (2) and (3), the nature of the surfactant and oil is characterized by the numerical values of the terms Cc and *EACN*, respectively. In all other cases, *EACN* must be determined experimentally. The constant *k* ≈ 0.15 scales the *EACN* for a fair sum with other terms. The influence of ionic strength enters through the salinity (*S*) of the system expressed as the grams of NaCl in 100 mL of water. The logarithmic relation between salinity for ionic surfactants is consistent with the fact that in these kinds of systems, zero salinity may not occur since the surfactant itself influences the ionic strength.

For this reason, when the NaCl concentration in water is less than 1% in weight, correction is required considering the non-negligible surfactant concentration. Otherwise, the salinity contributes linearly for nonionic amphiphiles, which are less sensitive to changes in ionic strength. The opposite sign in the correction for temperature in the case of ionic and nonionic surfactants accounts for the opposite morphology of the mEs observed when crossing the balance temperature; *c* ≈ 0.01 °C^−1^ < *c*’ ≈ 0.06 °C^−1^.

Recently, the term *HLD* has been associated with the spontaneous curvature of the interfacial film H_0_ normalized by its thickness *l* [149,150]:

(4) $$HLD\sim -H_{0}l$$

Equation (4) shows that a positive *HLD* corresponds to *H*_0_ < 0 (*w*/*o*), a negative *HLD* corresponds to *H*_0_ > 0 (*o*/*w*), and a null *HLD* indicates a balanced mE (*H*_0_ ≈ 0). The term accounting for the surfactant hydrophobicity is known as the surfactant characteristic curvature *Cc*. A comprehensive database of *Cc* and *EACN* values can be found in several papers [146,149,151,152,153,154,155], which allows for the prediction of the *HLD* (*H*_0_) of various mEs.

At a specific temperature, if the mE is balanced by a salinity *S* = S*, then the evolution of the film’s spontaneous curvature can be predicted by changing the salinity according to the following:

(5) $$HLD=-H_{0}l=ln\left(\frac{S}{S^{*}}\right)$$

This approach also works for mixtures of oils and surfactants of unknown *EACN* and *Cc* if their composition remains constant during the salinity scan.

Several studies have shown that the *HLD* concept can be used to design surfactant formulations that exhibit optimal performance in EOR applications [156,157]. The *HLD* concept has also been shown to be effective in the design of surfactant-polymer systems for EOR applications, exhibiting excellent performance in reducing interfacial tension and increasing oil recovery in core flood experiments [158]. In a study carried out by Nguyen et al. [131], the *HLD* equation was used in the characterization and selection of surfactants suitable for EOR. They determined the *Cc* value of surfactants which generated *HLD* values between −0.025 and 0.5, which brought about a balanced system of C14 oil at a temperature of 25 °C and the desired salinity.

In general, the *HLD* concept is a promising approach for the design of surfactants and surfactant–polymer systems for EOR applications. It can be said that *HLD* is more effective than HLB in the choice and design of a suitable surfactant for EOR. In addition, the choice between *HLD* and HLB should be based on the specific objectives of the surfactant characterization and the complexity of the system under investigation. Apart from offering greater flexibility and predictive capability in comparison to HLB, *HLD* is well suited for complex systems, can provide insights into the phase behavior of surfactant systems, and can be applied to a wide range of surfactant types, including non-ionic, anionic, cationic, and amphoteric surfactants, and it also takes into account interactions between surfactants and other components in a formulation, such as co-surfactants and electrolytes. While *HLD* offers these advantages, it is important to note that its application may require more sophisticated equipment and computational tools compared to the simpler HLB method. Further research is also needed to address the limitations of the *HLD* concept and to fully understand the factors that influence the performance of surfactants in EOR applications.

### 5.3. Characterization Techniques to Evaluate the Quality and Performance of Biosurfactants in EOR

Surfactants enhance oil recovery by reducing interfacial tension, altering wettability, and mobilizing oil displacement via emulsification [128]. The emulsification of the oil reduces its viscosity, thereby enabling its displacement from the reservoir rocks. Parameters like salinity and temperature are known to affect the efficiency of surfactants in EOR [159]. All these factors are important in understanding how to apply the surfactants in EOR processes, and the performance of the surfactants will be based on how well they are able to achieve the desired outcome of oil recovery. It is important to optimize the surfactant formulation, salinity levels, and other reservoir conditions to achieve the desired IFT reductions and microemulsion formation. The understanding of the complex interactions between salinity, crude oil viscosity, and surfactant behavior is crucial for effective oil recovery and enhanced oil production in different reservoir environments.

**Figure 5:**
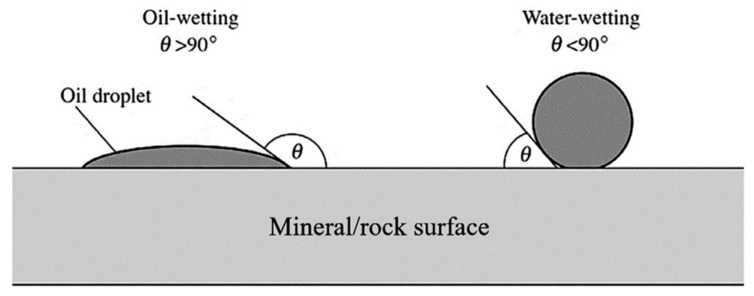
Surfactant alteration of the wettability of reservoir rock system. Reprinted from Ref. [160]. Copyright (2024), with permission from Elsevier.

Testing the effect of salinity and crude oil viscosity on the interfacial tension (IFT) reductions, wettability, and formation of microemulsion is a very effective way of evaluating the quality and performance of biosurfactants in EOR. Several techniques exist for testing IFT reduction, microemulsion formation, and wettability modification. In particular, wettability can be evaluated by a method called the contact angle technique. The contact angle (CA) is the angle formed between the intersection of the liquid–solid and liquid–vapor interfaces. This angle can be determined by evaluating the tangent line to the contact point along the liquid–vapor interface in the droplet profile (see Figure 5). The CA measurement is based on the surface tension of the liquid. In a liquid, surface molecules are not balanced by neighboring molecules in all directions, resulting in an inward pressure that creates an internal pressure. The liquid minimizes its surface free energy by contracting its surface area. When the solid surface is more affine to the liquid, the liquid spreads more on the solid surface (as shown on the left side of Figure 5). Conversely, when the affinity between the two phases decreases, the liquid beads on the solid (as shown on the right side of Figure 5). Therefore, high wettability is achieved when small CAs are measured, whereas low wettability is observed when CAs are large.

Regarding IFT reduction and microemulsion formation, there are several methods that can be used to test the effect of salinity and crude oil viscosity on the interfacial tension (IFT) reductions and formation of microemulsion. Here are a few commonly used methods:

- *Pendant drop method.* In this method, a small drop of one liquid (e.g., water) is suspended from the end of a needle or pipette, and then the other liquid (e.g., crude oil) is slowly added drop by drop until the two liquids meet at the interface. The interfacial tension can be calculated from the shape of the drop using the Young–Laplace equation. This method can be used to measure the IFT between two immiscible liquids and to test the effect of different parameters, such as salinity and crude oil viscosity, on the IFT. The pendant drop method is a straightforward technique that can provide accurate measurements of IFT with a high degree of precision. The shape of the droplet is analyzed using digital imaging techniques, and the IFT is calculated using the Young–Laplace equation. The droplet should be large enough to ensure accurate measurement of the dimensions but small enough to minimize the effects of gravity. The method is typically carried out at room temperature and atmospheric pressure. One limitation of the pendant drop method is that it requires specialized equipment and expertise to set up and perform.
- *Spinning drop method.* This method is similar to the pendant drop method, but the sample is rotated at a constant speed to minimize gravitational effects on the shape of the drop. This method can be used to measure the IFT between two immiscible liquids and to test the effect of different parameters, such as salinity and crude oil viscosity, on the IFT. The spinning drop method is a modified version of the pendant drop method that is performed under low gravity conditions, which can be achieved using a centrifuge or a drop tower. The method is particularly useful for measuring very low values of IFT, which cannot be accurately measured using the pendant drop method under normal gravity conditions. The spinning drop method is also more sensitive to small changes in IFT compared to the pendant drop method. However, the method requires specialized equipment and expertise to set up and perform and can be affected by various experimental parameters, such as the rotation rate and the size of the droplet [161].
- *Phase behavior experiments.* Phase behavior experiments involve preparing mixtures of two immiscible liquids, such as crude oil and water, with different concentrations of surfactant and/or different salinity levels. The mixtures are typically stirred and heated to allow equilibration, and the resulting phases are then observed and characterized. The phase behavior can be analyzed using various techniques, such as visual inspection, optical microscopy, and turbidity measurements. The IFT between the two liquids can also be measured using techniques such as the pendant drop or spinning drop method. Phase behavior experiments can provide valuable information on the effect of surfactant concentration, salinity, and other parameters on the formation of microemulsions and can help to optimize the design of EOR processes [162].
- *Microemulsion titration method.* In this method, a surfactant is added to the two liquids, and the mixture is titrated with a third liquid (e.g., an alcohol or an amine), while the IFT is monitored. The surfactant concentration can be adjusted to minimize the IFT, and the concentration at which the minimum IFT occurs can be used to determine the optimal surfactant concentration for forming a stable microemulsion. This method can be used to test the effect of different parameters, such as salinity and crude oil viscosity, on the formation of microemulsions. It is important to note that the microemulsion titration method is a technique for determining the optimal concentration of surfactant needed to form a stable microemulsion and not a method used to measure IFT in oil–water systems. The microemulsion titration method involves adding a surfactant to a mixture of two immiscible liquids (coarse emulsion), and then titrating the mixture with a third liquid (a cosurfactant which is usually a medium-chain-length alcohol) to form a stable microemulsion while simultaneously measuring the IFT. The concentration of surfactant is adjusted to minimize the IFT, and the concentration at which the minimum IFT occurs is taken as the optimal surfactant concentration for forming a stable microemulsion. The microemulsion titration method is useful for studying the effect of surfactant concentration, salinity, and other parameters on the formation of microemulsions, and can help to optimize the design of EOR processes. One limitation of the method is that it requires careful selection of the titrant liquid and the surfactant system to ensure accurate and reliable results [163].

## 6. Use, Application, and Effectiveness of Biosurfactants in EOR Processes

In general, biosurfactants have proven to be effective in increasing oil yield in secondary and tertiary oil recovery operations. Biosurfactants have been deployed to great use in studies that provided promising results for the future of eco-friendly EOR. A study carried out by Lal et al. [164] involved the investigation of the potential of by-products of a microbial consortium of anaerobic bacterial strains in enhancing oil recovery from oil reservoirs under the temperature range of 70–90 °C. The results showed an increase in the efficiency of sweeping crude oil from oil-bearing poles of rock formation and also brought about an oil-improving recovery process. In another study carried out by Arima et al. [165], surfactin, a lipopeptide synthesized from the *Bacillus* strain, was characterized and investigated as a potential EOR biosurfactant due to its high surface activity and low critical micelle concentration (CMC) in comparison to synthetic surfactants. The results demonstrated a lowered surface tension and a reduced interfacial tension against a hexadecane concentration of 1 mN/m. It was also shown to be stable under high pH conditions, high-salinity conditions, and high temperatures.

The results of several studies [30,166,167,168] show interfacial tension values of less than 0.001 mN/m between water and oil via the use of lipopeptides. However, the drawback that comes with the application of lipopeptides in EOR is relatively low yield and high manufacturing costs when they are produced via fermentation; thus, they are hardly ever applied in field-scale EOR operations.

Blesic et al. [116] investigated a class II hydrophobin HFB II obtained from *Trichoderma reesei* as a biosurfactant for EOR purposes. Their study indicated that HFB II does not seem to be a promising candidate for EOR as high-salinity and high-temperature conditions, which are typical of oil recovery situations, appear to have a negative effect on its surfactant potential. Regardless of this disadvantage, the hydrophobin HFB II was still able to form films and relatively strong emulsions in synthetic seawater up to 70 °C, and its emulsifying power was comparable to that of other surfactants. However, the performance of the hydrophobin HFB II in EOR can be improved if it is applied in combination with an appropriate co-surfactant, such as a medium-chain alcohol. This will facilitate microemulsion formation and reduce the tendency to form a rigid gel-like structure. A 2021 study carried out by Nafisifar et al. [127] indicated a 7.9% increase in oil recovery using a biosurfactant obtained from linseeds. This biosurfactant reduced the surface tension by 96%, the interfacial tension by 59%, and the wettability was also improved by 58%. The biosurfactant also proved to be stable at high salt concentrations and, in general, has a great potential for EOR applications.

Another recent study was carried out by Tackie-Otoo et al. [169] investigating the EOR potential of two biosurfactants synthesized from N-lauroyl sarcosin (NLS) and lauroyl glutamic acid (LGA). Although NLS proved to perform better than LGA in general, LGA yielded more stable emulsions. NLS was more surface active than LGA, and this was shown to be because of the additional carboxylate attached to the head group of LGA, which worsened its surface-active potential. NLS also had better emulsifying power due to its superior interfacial tension reduction capability. Both surfactants showed high salt tolerance, and they both achieved significant oil recovery after waterflooding, with NLS improving oil recovery by 43% and LGA by 25%. Therefore, NLS and LGA prove to be superb alternatives to conventionally deployed EOR surfactants due to their satisfactory performances coupled with their environmentally benign nature.

Domdi et al. [170] demonstrated the effectiveness of a biosurfactant produced by *Pseudomonas aeruginosa* in reducing interfacial tension and improving oil recovery in sand-packed columns. The biosurfactant was found to be more effective than anionic surfactant sodium dodecyl sulfate (SDS) and showed 68.53 ± 3.07% of oil recovery in the sand pack column under saline conditions, and it was also shown to be biodegradable and non-toxic. This study reported that the purified biosurfactant PU1 showed a reduction in the surface tension of water from 70.23 mN/m to 29.77 mN/m, at a concentration of 30 mg/L, which can be correlated to the IFT value. Another example of a successful application of a novel biosurfactant in EOR is the use of rhamnolipids produced by *Pseudomonas aeruginosa* in low-permeability reservoirs. The rhamnolipids were found to be effective in reducing interfacial tension and improving oil recovery, and they were also shown to be biodegradable and non-toxic [171]. Other types of novel biosurfactants have also been investigated for their potential applications in EOR. For example, Atta et al. [172] synthesized a series of novel surfactants based on natural amino acids, and they demonstrated the effectiveness of these surfactants in reducing interfacial tension and improving oil recovery in sand-packed columns. 

Despite the promising results obtained with novel biosurfactants in EOR, there are still some challenges that need to be addressed. For example, some of the IFT values obtained after application of the biosurfactants are not as low as some of the values obtained using conventional chemical surfactants for EOR, meaning the efficiency of some biosurfactants has to be improved. The production cost of some biosurfactants is also higher than that of traditional surfactants, which may limit their widespread adoption in EOR operations. In addition, the performance of these biosurfactants may be affected by environmental factors such as temperature, pressure, and salinity, which can vary significantly from one reservoir to another. Some additional case studies on the application of novel biosurfactants in EOR include the use of saponin-based surfactants investigated as potential green alternatives to traditional surfactants for EOR. A study conducted by Khayati et al. [173] investigated the performance of pure saponin, a non-ionic surfactant, for EOR in sandstone and carbonate reservoirs. The key results of this study include the effectiveness of saponin in reducing IFT between oil and water and altering the wettability of reservoir rock. The study also found that saponin was able to improve oil recovery in core flood experiments.

Alkyl poly glucosides (APGs) are another type of biosurfactant that has been investigated for use in EOR at high-temperature and high-salinity environments. The study by Li et al. [31] investigates the effectiveness of alkyl poly glycoside (APG), a plant sugar-derived green biosurfactant with excellent interfacial activity, emulsified ability, foaming performance, and wettability, in enhancing heavy oil recovery at high-temperature and high-salinity condition. The study found that APG had excellent interfacial activity and emulsification properties among all the surfactants tested. The interfacial activity and emulsification properties of APG did not decrease and even improved along with the increasing temperature or salinity. The incremental oil recovery using APG at 90 °C and the salinity of 30 g/L can reach 10.1%, which is nearly two times higher than that of common EOR surfactants. These results indicated that APG is an efficient surfactant for enhancing heavy oil recovery at high-temperature and high-salinity conditions.

In a recent study by Bachari et al. [109], the development of improved surfactants for enhanced oil extraction has superior capabilities while being environmentally friendly and capable of strong operational tolerances to pH, salinity, and temperature. The key results of this article include the finding that numerous biosurfactants synthesized from vegetable oils and other plant-based materials matched or exceeded the capabilities of conventional synthetic surfactants. Plant-based zwitterionic surfactants are reported to have strong interfacial reduction values and operational tolerances [110].

Biosurfactant-producing and oil-degrading *Bacillus subtilis* strains demonstrate a potential application in enhancing oil recovery sand pack columns. For instance, Gudiña et al. [16] investigated the use of biosurfactant-producing and oil-degrading *Bacillus subtilis* strains to enhance oil recovery in laboratory sand pack columns. The study found that indigenous *Bacillus subtilis* strains produced biosurfactants inside the columns and also degraded the long-chain n-alkanes and reduced oil viscosity in porous media. Both processes led to an improvement in the oil recovery.

Surfactant formulation has been employed for green EOR. In their study, Al-Ghamdi et al. [174] investigated the use of biosurfactant formulations for EOR and their effectiveness in reducing interfacial tension and improving oil recovery. The study found that a blend of rhamnolipid, APG, and lecithin was considered a possible formulation to investigate the efficacy of these biosurfactants in sandstone reservoirs based on phase behavior studies, interfacial tension measurement, and core-flooding experiments. The study found that all three blends performed well in the phase behavior study, resulting in stable middle-phase microemulsions, whereas only one formulation had an ultra-low IFT reduction between oil and brine. Consequently, this formulation performed the best of the three, with a tertiary recovery of 24% and a total recovery of approximately 70%. These results suggest that while other blends had suitable EOR potential, the third formulation is considered a more appropriate candidate for chemical EOR with a preference for biosurfactants (rhamnolipids) application.

Lignin-based surfactant can be utilized as a green surfactant in EOR. The study by Ganie et al. [175] aimed to determine the formulations of the lignin-based surfactant for EOR applications and to determine the oil recovery performance of the formulated surfactants through surfactant flooding. The lignin-based surfactants were formulated by mixing the lignin with the amine (polyacrylamide or hexamethylenetetramine) and the surfactant sodium dodecyl benzene sulfonate in a 20,000 ppm NaCl brine. IFT of the formulated lignin-based surfactant is measured at ambient temperature using the spinning drop method. The displacement experiments were conducted at room temperature in glass bead pack holders filled with glass beads saturated with paraffin and brine.

Additionally, lignin nanoparticles (LNPs) have shown to be highly efficient, recyclable surfactants for EOR. Gao et al. [176] reported on the use of LNPs as Pickering surfactants obtained from enzymatic hydrolysis of lignin powders to apply in EOR. The interfacial activity of LNPs prepared this way is greatly improved, which substantially promotes its emulsification ability. The study shows that kerosene emulsified with different concentrations of LNPs can form stable Pickering emulsions, and the emulsions can be stored for up to 6 months. In addition, due to the pH-responsive character of lignin, rapid oil–water separation can be achieved by alkali demulsification. This enables the reuse of lignin suspension under pH control, which provides a new platform for the application of green and low-cost flooding, employing LNPs in EOR. In addition to the aforementioned case studies of biosurfactants in EOR, more information on other cases involving the application of biosurfactants for EOR purposes, the testing conditions, and the respective IFT values obtained can be found in Table 3.

The aforementioned case studies all demonstrate the potential of novel biosurfactants as alternatives to traditional surfactants for EOR. In most cases, the surfactant was found to be effective in reducing interfacial tension, altering wettability and/or facilitating emulsification thus improving oil recovery. In terms of specific types of biosurfactants, protein-based surfactants showed promise in several studies, with oil recovery efficiencies ranging from 21% to 25%. Lignin-based, betaine-based, and polysaccharide-based surfactants also showed suitable performance, with oil recovery efficiencies ranging from 22% to 28.3%. However, it is important to note that the performance of these surfactants can vary depending on the specific conditions of the reservoir and the type of oil being extracted. For example, some surfactants may be more effective in low-salinity or high-temperature conditions. Therefore, further research is needed to fully optimize the production and application of these surfactants for different types of reservoirs and oils.

In addition, it is important to consider the economic feasibility of using these novel biosurfactants compared to traditional surfactants. While they may be more environmentally friendly, they may also be more expensive to produce and may require additional processing steps. Therefore, a cost–benefit analysis should be performed before deciding on the use of these surfactants in EOR. Overall, while these case studies demonstrate the potential of novel biosurfactants for EOR, further research and optimization are needed to fully realize their benefits and to determine their economic feasibility. The use of these biosurfactants in EOR operations is a promising approach that has the potential to improve the sustainability of oil recovery processes. Further research is needed to optimize the production and performance of these surfactants and to identify the most suitable applications and conditions for their use in EOR operations.

### Challenges and Limitations of Implementing Biosurfactant-Based Technologies in EOR

Although the use of biosurfactants in EOR offers many benefits, it is important to keep in mind some factors, such as their cost and scalability. In some cases, the synthesis of biosurfactants can be quite expensive when compared to traditional chemical surfactants. Biosurfactants such as zwitterionic surfactants [51], specialized production and processing techniques such as submerged fermentation [107] have high costs, and this can be a stumbling block in the advancement of biosurfactant-based technologies in EOR. Scaling up production in order to meet large-scale EOR operation requirements can further drive up the cost of biosurfactant production. The development of cheaper techniques and raw materials in addition to yield optimization are important in mitigating the high costs associated with some groups of biosurfactants.

The intricate nature of biosurfactants also makes them sensitive to environmental conditions such as salt concentration, temperature, and pH [179]. Oil reservoir conditions could be harsh, and ensuring the stability and compatibility of biosurfactants will increase their potential for EOR applications. The biosurfactants also have to be compatible with the existing equipment, infrastructure, and other polymers used in the oil recovery operations. This is to prevent reduced performance and undesired reactions during EOR. It is also worth noting that each oil reservoir has its specific geological and petrophysical properties. Parameters such as interfacial properties, rock wettability and permeability influence biosurfactant effectiveness in EOR. This should be kept in mind in the design of biosurfactants and the injection strategies in order to optimize their performance. The HLD concept is a very important tool that should be applied in the design and choice of EOR surfactants as it takes into account several key parameters that influence surfactant–oil–water systems such as oil reservoirs [180]. Another important point to consider is that although biosurfactants have been demonstrated to be promising in laboratory and small-scale field trials, there is still not a lot of extensive field application data unlike traditional chemical surfactants. They need to be compared with conventional surfactants on larger scales and real-world application studies in order to validate their performance.

## 7. Future Perspectives

Biosurfactants have a variety of applications, and the future holds so many possibilities regarding their applications. In EOR, to be precise, more steps need to be taken in order to optimize the use of biosurfactants to improve oil recovery. In the short term, the development process should increase the production of biosurfactants with hydrophilic–lipophilic balance (HLB) values between 8 and 12 in order to favor the formation of oil-in-water emulsions, which are preferable for EOR applications. This HLB recommendation, however, applies only to ethoxylated and non-ionic biosurfactants. Due to some of the advantages *HLD* has over the HLB concept, such as higher predictive capability, better suitability for complex systems with co-surfactants, and applicability across a wide range of surfactant types, more work should be performed using *HLD* in establishing a standard classification of surfactants on the basis of their suitability for different EOR conditions. *HLD* should also be used in conjunction with mathematical modeling to simulate surfactant behavior in different scenarios. Using *HLD* to tailor surfactants to specific applications and effects will save time and resources and also greatly improve the efficiency of EOR operations.

Regarding microbial fermentation processes in biosurfactant production, cheaper raw materials and substrates, waste valorization, and better extraction and purification techniques need to be researched, which will improve methods and optimize production yield. Biosurfactants that have excellent properties for EOR applications, such as zwitterionic surfactants and Gemini surfactants, should be more thoroughly researched, and better, cost-effective methods for their synthesis be brought to light. This will improve the efficiency of biosurfactants in large-scale EOR operations. Since there are more oil reserves in carbonate reservoirs and cationic surfactants need to be used in that oil recovery context, cheaper ways of producing these surfactants from cheap, natural, renewable sources to replace conventional chemical surfactants need to be further investigated. In the long term, even after the successful use of biosurfactants in EOR operations, continuous monitoring and assessment of reservoir conditions and biosurfactant production outcomes are fundamental in order to establish a sense of reliability in biosurfactants, their efficiency, and practical feasibility in oil recovery.

Furthermore, the environmental impact of biosurfactants needs to be carefully evaluated. While they may be biodegradable and derived from renewable resources, their production can still have an environmental impact. Life cycle assessments of biosurfactants in the long term are needed to ensure their sustainability and environmental benefits. In addition, as some biosurfactants cannot be used alone in EOR without the use of co-surfactants, the adherence of the combinations and formulation of these surfactants to standard policy, legislation, and regulatory standards need to be assessed. Their environmental impact should be studied.

To overcome the current challenges in biosurfactant applications in EOR operations, future research directions should focus on several critical aspects relevant to the biosurfactants:

- Developing novel biosurfactant-producing microorganisms and biosurfactant types that can adapt to harsh reservoir conditions, such as high salinity, temperature, pressure, or acidity. This could involve genetic engineering, metabolic engineering, or synthetic biology approaches to enhance the biosurfactant production and performance of microorganisms.
- Exploring the synergistic effects of biosurfactants with other EOR agents, such as polymers, nanoparticles, gases, or enzymes. This could involve designing and testing novel biosurfactant-based formulations or systems that can improve the oil recovery efficiency and reduce the operational costs and environmental impacts of EOR.
- Investigating the mechanisms and kinetics of biosurfactant interactions with oil, water, rock, and other reservoir components. This could involve using advanced analytical techniques, such as spectroscopy, microscopy, rheology, or chromatography, to characterize the physicochemical and biological properties and behaviors of biosurfactants in the reservoir system.
- Developing reliable and robust models and methods for predicting and optimizing the biosurfactant performance and efficiency in the reservoir. This could involve using artificial intelligence, machine learning, or data mining techniques to analyze and integrate the data from laboratory experiments, numerical simulations, and field trials.
- Evaluating and mitigating the environmental impact and sustainability issues of biosurfactant production and injection in EOR. This could involve conducting a life cycle assessment, environmental risk assessment, or social impact assessment of biosurfactant application in EOR. It could also involve developing strategies for reducing energy and water consumption, minimizing chemical leakage or spillage, enhancing biodegradability or recyclability, or improving the social acceptance or awareness of biosurfactant applications in EOR.

Addressing these research gaps will surely advance the knowledge and technology of biosurfactants in EOR.

## 8. Conclusions

EOR is an important technique used in the oil and gas industry to recover more oil from reservoirs after primary and secondary recovery methods have been exhausted. The process involves injecting different chemicals or gases into the reservoir to reduce the surface tension between the oil and the rock surface, making it easier for the oil to flow towards the production wells. This technique has been in use for several decades, and a wide variety of chemical agents have been used, including surfactants. However, traditional surfactants used in EOR are often derived from petroleum, which can have negative environmental impacts, such as toxicity and persistence in the environment. Biosurfactants on the other hand, are derived from renewable resources and are biodegradable, making them potentially more sustainable and environmentally friendly.

These bio-based surfactants can be derived from a variety of sources, including plant-based sources, and can be synthesized using environmentally friendly methods. They have shown promising results in laboratory experiments and small-scale field trials, and they have the potential to be used in commercial applications in the future. One of the major challenges in the development of bio-based surfactants for EOR is their cost-effectiveness. Some are rather cheap to synthesize while others require feedstock and specialized equipment and methods amounting to higher production costs. Although new studies proposing cheaper ways to synthesize these surfactants are becoming increasingly common, biosurfactants are still sometimes more expensive than traditional surfactants derived from petroleum. This can be a barrier to their adoption in commercial applications. However, advancements in technology and scale-up production methods is helping to reduce costs and is expected to keep doing so for the foreseeable future. Another challenge is the compatibility of biosurfactants with reservoir conditions. Surfactants are sensitive to temperature, pressure, and salinity, and their performance can be affected by these factors. The development of biosurfactants that can perform well under a wide range of reservoir conditions is crucial for their commercial success.

In conclusion, the use of biosurfactants in EOR shows promise as a potential alternative to traditional petroleum-based surfactants. However, more research is needed to develop cost-effective, compatible, and sustainable biosurfactants that can perform well under a wide range of reservoir conditions. The future holds great promise for the use of these surfactants in EOR, but their commercial viability will depend on overcoming a few challenges. The development and adoption of biosurfactants in EOR can contribute to a more sustainable and environmentally friendly oil and gas industry.

## Figures and Tables

**Table 3:** Biosurfactants, their producing organisms/source, the EOR conditions, and their respective IFT measurements. Adapted from [128,177,178].

Microorganism/Source	Surfactant	Oil Phase	Salinity (NaCl) wt%	Temp (°C)	Surfactant Concentration (wt%)	IFT (mN/m)
*Bacillus subtilis* R14914	Surfactin	Crude oil	-	-	-	0.2
*Bacillus subtilis* 22.2	Surfactin	Crude oil	-	25	-	0.12
*Bacillus licheniformis* W16	Lichenysin A	Crude oil	-	60	-	15.06
*Pseudomonas aureginosa* HAK01	Rhamnolipid	Crude oil	-	25	-	2.50
Jatropha oil	Sodium Methyl Ester Sulphonate (SMES)	Crude oil	2	50	0.01–1	0.079
Castor oil	Sodium Methyl Ester Sulphonate (SMES)	Crude oil	1–5	29	0.1–0.8	0.034
Castor oil	Polymeric Sodium Methyl Ester Sulphonate (PMES)	Crude oil	1–5	29	0.1–0.5	0.066
Waste cooking oil	PFAPMB (Zwitterionic Surfactant)	Crude oil	NaCl/Divalent ions	50	0.001–0.05	0.0016
Waste cooking oil	SPODP (Zwitterionic surfactant)	Crude oil	CaCl_2_/NaCl	50–100	0.05–0.3	0.003
Soapwort plant extract	Non-ionic surfactant	Crude oil	-	80	0.075–0.035	0.834
*Glycyrrhiza glabra* plant extract	Saponin	Kerosene	-	25	1–8	6.5
*Pseudomonas sp.*	Rhamnolipid	Crude oil	-	-	-	0.080
*Pseudomonas sp.*	Rhamnolipid	Isooctane	-	-	-	0.285

## Data Availability

Not applicable.
